# Protein arginine methylation and ubiquitination: A prominent crosstalk and its functions in cancer

**DOI:** 10.1016/j.gendis.2025.101997

**Published:** 2025-12-19

**Authors:** Jiawen Zhou, Ao Zhang, Jiuling Zhu, Fei Tang, Ziyang Yuan, Wenlong Ma, Qi Wang, Jun Lu, Shu Li, Zhongwei Li

**Affiliations:** a Laboratory of Epigenetic Regulation in Molecular Medicine, School of Basic Medical Sciences, Wannan Medical University, Wuhu, Anhui 241002, China; b School of Life Science, Northeast Normal University, Changchun, Jilin 130024, China; c Department of Physiology, School of Basic Medical Sciences, Health Science Center, Shenzhen University, Shenzhen, Guangdong 518060, China; d Department of Pathophysiology, School of Basic Medical Sciences, Wannan Medical University, Wuhu, Anhui 241002, China; e Anhui Province Key Laboratory of Basic Research and Transformation of Age-related Diseases, Wannan Medical University, Wuhu, Anhui 241002, China

**Keywords:** Arginine methylation, Cancer, Crosstalk, PRMTs, Ubiquitination

## Abstract

Protein arginine methyltransferases (PRMTs) catalyze the formation of arginine methylations in histones and nonhistone proteins. PRMT family members strongly affect the malignant progression of cancer. Recently, an increasing number of studies have shown that the interactions of protein arginine methylation and ubiquitination play crucial roles in various essential biological processes related to cancer, such as the DNA damage response (DDR), protein stability, immune escape and signal transduction, which significantly influence cancer progression. This article presents an overview of the mechanisms by which the crosstalk between arginine methylation and ubiquitination impacts cancer. Moreover, we explore future research directions related to arginine methylation and ubiquitination crosstalk in cancer treatment. The goal is to provide a theoretical foundation and potential applications for related basic research and the development of anticancer drugs.

## Introduction

Cancer treatment continues to pose a significant challenge in the medical field, primarily due to the high degree of similarity and the pathological differences between cancer cells and normal cells in key regulatory mechanisms.^1^^,^^2^ Recent studies have demonstrated that arginine methylation influences protein function and interactions, contributing to the epigenetic regulation of cancer.^3^ Moreover, the ubiquitination system modulates the stability and activity of target proteins through specific labeling.^4^ Notably, these two posttranslational modification systems not only play critical roles in cancer development but also form a complex interactive network: methylation can alter the degradation efficiency of oncogenes by affecting the activity of ubiquitinase complexes, while the ubiquitination process can inversely regulate the stability of protein arginine methyltransferases (PRMTs).^5^^,^^6^ This dynamic interaction is pivotal in key pathological processes, such as remodeling of the cancer microenvironment and the development of treatment resistance.^7^, ^8^, ^9^, ^10^, ^11^ Therefore, a deeper understanding of their molecular dialog mechanisms will provide a theoretical foundation for the development of new combined therapeutic strategies.^12^^,^^13^

Recent studies have revealed a complex regulatory network among the posttranslational modification systems of proteins, with a particular emphasis on the dynamic interaction between arginine methylation and ubiquitination.^14^, ^15^, ^16^ Research indicates that varying patterns of methylation can influence the recognition and recruitment efficiency of ubiquitin ligases, thereby bidirectionally modulating the stability and functional activity of key regulatory factors.^17^^,^^18^ At the epigenetic level, specific methylations can alter the spatial conformation of chromatin complexes, thereby affecting the efficiency of the DNA damage response system.^19^, ^20^, ^21^ Similarly, synergistic modification mechanisms involving methyltransferases and deubiquitinases can serve as a molecular basis for the sustained activation of oncogenic signaling pathways.^22^, ^23^, ^24^, ^25^ This multidimensional interaction network of modifications offers a novel perspective for understanding the mechanisms underlying cancer development.

However, reviews addressing the interplay between arginine methylation and ubiquitination in cancer are limited. A more comprehensive understanding of this cross-regulatory network of posttranslational modifications is essential for the development of more effective anticancer therapies.

## Classification, structure and function of PRMTs

### Classification and catalytic mechanism of PRMT enzymes

**Figure 1:**
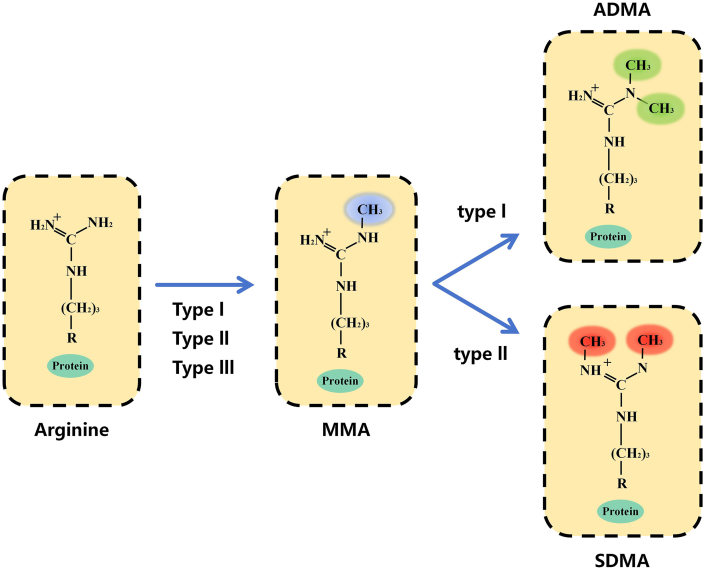
Classification of PRMT enzymes. PRMTs, which act on the arginine residues of proteins, are primarily categorized into three types: Type I, Type II, and Type III. Type I PRMTs (PRMT1, PRMT2, PRMT3, PRMT4, PRMT6, and PRMT8) catalyze the ADMA of the substrate protein. Type II PRMTs (PRMT5 and PRMT9) catalyze the SDMA of the substrate protein. Type III PRMTs, which include only PRMT7, catalyze the MMA of the substrate protein.

Protein arginine methyltransferases (PRMTs) are classified into three types on the basis of their catalytic mechanisms and product specificity: Type I (PRMT1, 2, 3, 4, 6, 8), Type II (PRMT5, 9), and Type III (PRMT7) (Fig. 1).^26^^,^^27^ Type I enzymes catalyze asymmetric dimethylation (ADMA); type II enzymes catalyze symmetric dimethylation (SDMA); and type III enzymes possess tandem dimethyltransferase domains that enable continuous single-methylation (MMA) on specific substrates.^28^^,^^29^

All PRMT family members contain conserved methylation transfer core domains, where the S-adenosylmethionine (SAM)-binding pocket precisely controls methylation efficiency through conformational changes.^30^^,^^31^ Type I enzymes contain β-helical propeller domains to recognize nuclear localization signals; type II enzymes form homodimers via α-helix bundles and assemble into heteromeric complexes with the chaperone protein MEP50; and type III PRMT7 contains tandem methylation transferase domains linked by flexible linker peptides, enabling continuous methylation.^32^, ^33^, ^34^ All PRMTs possess an acidic catalytic triad at their active sites, which determines the specificity of their substrate methylation patterns.^35^^,^^36^ The catalytic process begins with the ε-amino group of the arginine residue binding to the methyl group provided by SAM.^37^ Multiple functional domains collaborate to precisely control the degree of methylation and the subcellular localization of the enzyme complex.^38^

### The multifaceted roles of PRMTs in cancer development

The PRMT family establishes a multidimensional regulatory network within the cancer microenvironment through arginine methylation.^21^^,^^27^ For example, at the epigenetic level, PRMT1 drives Epithelial–mesenchymal transition (EMT) in colorectal cancer (CRC) through the modification of histone H4R3me2a.^39^ PRMT5-mediated histone arginine methylation counteracts transcriptional repression by Polycomb repressive complex 2 (PRC2), thereby regulating the proliferation of leukemia cells. In terms of metabolic reprogramming.^40^ PRMT3 influences the mRNA stability of key enzymes involved in glycolysis in hepatic cancer.^41^ The loss of PRMT7 results in decreased expression of glycine decarboxylase, triggering glycine metabolic reprogramming and the production of methylglyoxal, which disrupts the growth of leukemia stem cells.^42^ Additionally, PRMT1 regulates the phase separation of autophagy-related proteins, contributing to chemotherapy resistance in gliomas.^43^ In renal injury models, PRMT1 mediates acute kidney injury induced by septicemia in mice through TGF-β1 growth factors and the IL-6 inflammatory signaling pathway.^44^ Therefore, PRMTs, a class of essential protein methyltransferases, play crucial regulatory roles in various biological processes associated with cancer development, including cell proliferation, apoptosis, invasion and metastasis, and angiogenesis. Collectively, these processes regulate malignant transformation and progression in a variety of cancers.^45^, ^46^, ^47^, ^48^, ^49^

## Protein ubiquitination in cancer

### E3 ligases and ubiquitination mechanism

**Figure 2:**
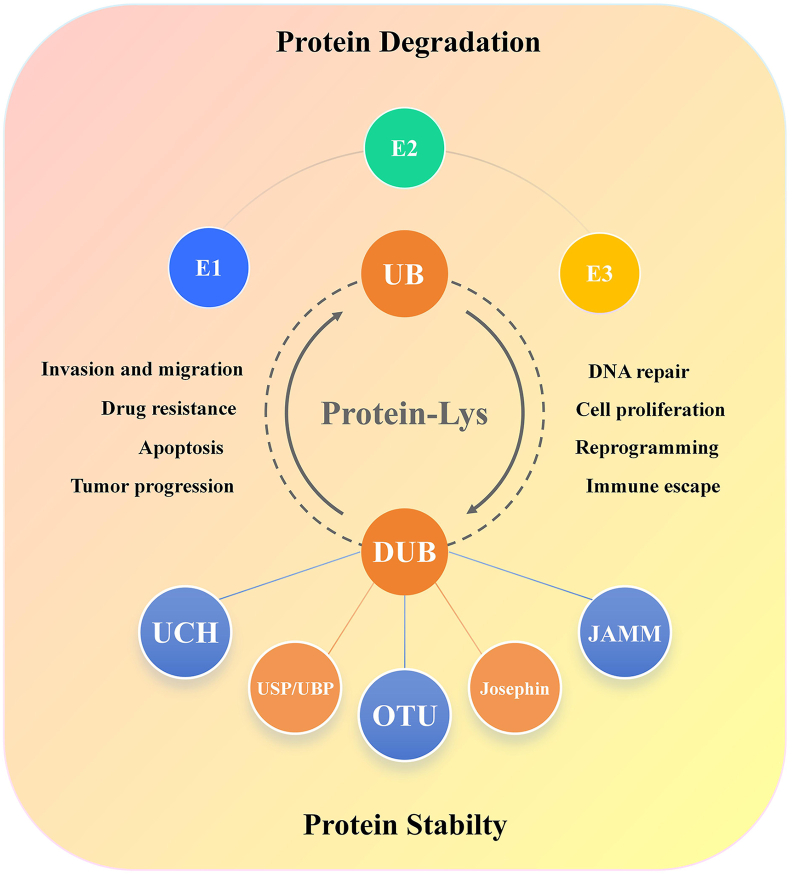
Ubiquitinase and deubiquitinating enzyme protein family members. The ubiquitination process regulates protein stability and is mediated by ubiquitinases and deubiquitinases. Ubiquitinases act on the lysine residues of proteins through a series of catalytic steps involving E1, E2, and E3 enzymes. Deubiquitinases are classified into five families on the basis of their structural domains and substrate specificity. These enzymes play crucial roles in various cellular processes and are closely associated with the development and progression of cancer.

Ubiquitination is a dynamic and reversible posttranslational modification of proteins that involves a three-step enzymatic reaction mediated by E1, E2, and E3 ubiquitin ligases (Fig. 2).^50^^,^^51^ This process covalently links the ubiquitin molecule to the target protein. E3 ligase families, such as the SCF complex, APC\C, and MDM2, possess specific recognition functions for their targets.^51^, ^52^, ^53^

Deubiquitinases (DUBs) reverse the ubiquitination process by hydrolyzing the isopeptide bond between ubiquitin and the target protein.^54^^,^^55^ These enzymes are categorized into six subfamilies: USP, UCH, OTU, MJD, ZUP1, and JAMM.^56^^,^^57^ DUBs play crucial roles in various cellular activities by regulating the ubiquitination and degradation of key cell cycle proteins, tumor suppressor proteins, and factors involved in signaling pathways.^58^^,^^59^

### Role of ubiquitination in cancer

Ubiquitination regulates the stability of key oncogenes and tumor suppressor genes, profoundly influencing the fundamental biological processes involved in cancer development.^60^^,^^61^ Recent studies have indicated that E3 ubiquitin ligases can be used to evaluate the tumor immune microenvironment and prognosis in hepatocellular carcinoma (HCC) patients.^62^ When cells detect DNA double-strand breaks (DSBs), the E3 ubiquitin ligase RNF168 rapidly ubiquitifies histone deacetylase 6 (HDAC6) to promote its degradation, restoring its interaction with H2A/H2A. X and enabling H2A/H2A. X ubiquitination.^63^ This facilitates the recruitment of DSB repair factors for DNA repair. In renal ischemia‒reperfusion injury models, TRIM35 interacts with p53-induced glycolysis and apoptosis regulator (TIGAR), promoting TIGAR polyubiquitination and degradation while inhibiting mitochondrial fusion.^64^ Additionally, hypoxia-inducible factor-1α (HIF-1α) escapes degradation in hypoxic microenvironments through the deubiquitinating enzyme USP28, driving tumor angiogenesis and metabolic reprogramming.^65^ K63 ubiquitination of programmed death ligand 1 (PD-L1) promotes lysosomal pathway degradation, while the deubiquitinating enzyme USP22 stabilizes its membrane localization, enhancing tumor cell immunorevasion.^66^

The dynamic equilibrium between ubiquitination and deubiquitination not only directly regulates cellular protein homeostasis but also reshapes the tumor microenvironment through complex signaling networks.^4^^,^^60^ For example, in the cell proliferation signaling pathway, the ubiquitination-mediated degradation of the tumor suppressor p53 by the E3 ubiquitin ligase MDM2 is a critical factor in maintaining cell cycle progression in cancer cells.^67^^,^^68^ Conversely, the deubiquitinating enzyme USP7 maintains delicate dynamic equilibrium by specifically removing ubiquitin chains from p53.^69^ With respect to antiapoptotic mechanisms, the E3 ligase XIAP inhibits the activity of caspase family proteins through ubiquitination, while USP9X maintains the stability of the antiapoptotic MCL1 protein via deubiquitination, thereby significantly increasing drug resistance in cancer cells.^70^^,^^71^ These multilevel and multifaceted regulatory mechanisms not only reveal the pivotal role of the ubiquitination system in tumor development but also provide a solid theoretical foundation and potential therapeutic targets for developing anticancer drugs targeting the ubiquitination system.^72^, ^73^, ^74^, ^75^

### Crosstalk between arginine methylation and ubiquitination in cancer

**Figure 3:**
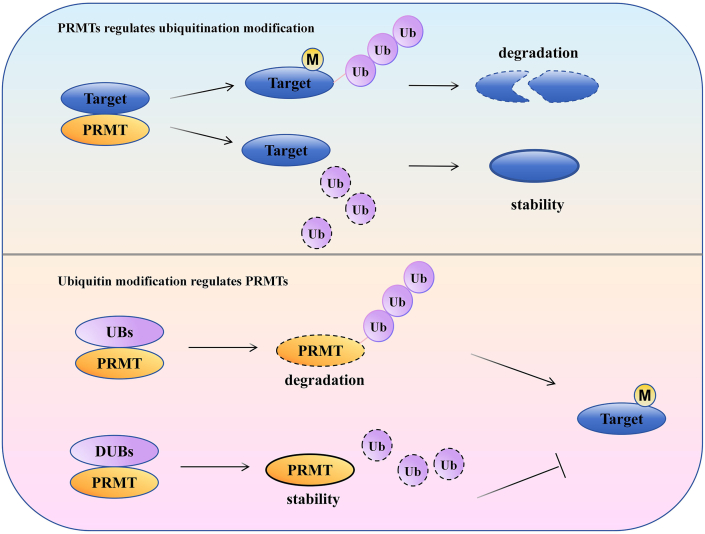
Crosstalk between PRMT enzymes and (de)ubiquitinases. The figure illustrates the primary interactions between PRMT enzymes and (de)ubiquitinases. In the upper section of the figure, PRMT enzymes modify the arginine methylation state of target proteins, which subsequently influences the (de)ubiquitinase modifications of these proteins. In the lower section, (de)ubiquitinases directly interact with PRMT enzymes, either by ubiquitinating or deubiquitinating them. This interaction alters the stability of PRMT enzymes and impacts the arginine methylation of downstream proteins.

**Figure 4:**
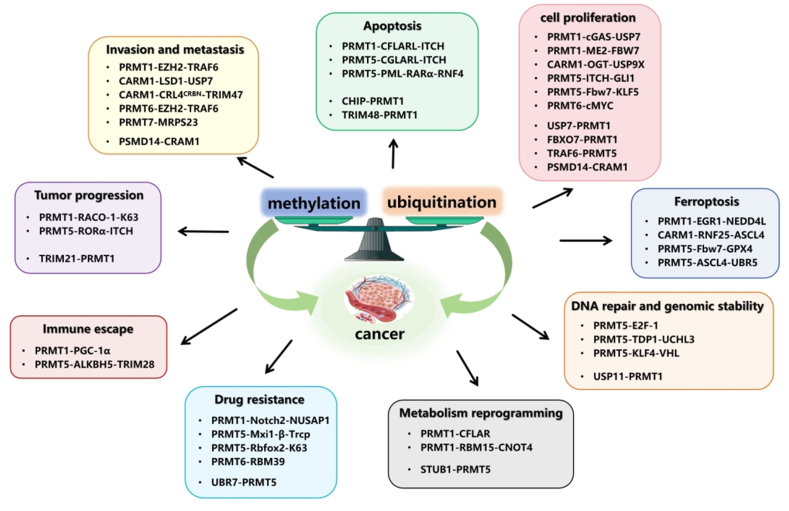
The impact of crosstalk between arginine methylation and ubiquitination on cancer development and malignant progression. Under normal physiological conditions, the crosstalk between methylation and ubiquitination maintains a dynamic equilibrium. When this balance is disrupted, it may trigger tumor development and promote disease progression. The crosstalk between arginine methylation and ubiquitination mediated by PRMT enzymes has multifaceted effects on tumors and plays a crucial role in eight key biological characteristics of cancer.

In recent years, research on the synergistic regulation of tumor-related mechanisms through protein arginine methylation and ubiquitination has significantly expanded (Table 1).^43^^,^^76^ These studies revealed that the dynamic interplay between epigenetic modification enzymes and the ubiquitin system constitutes the critical molecular basis for tumorigenesis.^77^^,^^78^ This “methylation-ubiquitination” regulatory network acts as a master switch controlling key protein functions during tumor metastasis, proliferation, and disease progression. As shown in Figure 3, it precisely regulates target protein ubiquitination status (through degradation or deubiquitination stabilization) through methylations, thereby altering protein stability, activity, or downstream signaling outputs to achieve precise control over tumor pathways. Conversely, ubiquitination directly modulates PRMT enzyme activity, influencing its regulation of downstream proteins and ultimately affecting overall tumor progression (see Fig. 4).

**Table 1:** PRMT-mediated methylation regulates the ubiquitination process.

PRMT enzyme	(De)ubiquitinating enzymes	Target protein	cancer	Functions	Ref
PRMT1	TRAF6	EZH2	BC	Invasion and migration	79
	USP7	cGAS	NSCLC	Cell proliferation	80
	FBW7	ME2	HCC	Cell proliferation	81
	ITCH	CFLARL	NSCLC	Apoptosis	82
	NEDD4L	EGR1	ALI	Ferroptosis	83
	\	CFLAR	HCC	Metabolism reprogramming	84
	CNOT4	RBM15	APL	Metabolism reprogramming	85
	NUSAP1	Notch2	GC	Drug resistance	86
	\	PGC-1α	NPC	Immune escape	87
	\	RACO-1	PANCAN	Tumor progression	88
PRMT4	USP7	LSD1	BC	Invasion and migration	89
	CRL4^CRBN^	TRIM47	HCC	Invasion and migration	3
	USP9X	OGT	NSCLC	Cell proliferation	90
	ACSL4	RNF25	CRC	Ferroptosis	91
PRMT5	GLI1	ITCH/NUMB	PANCAN	Cell proliferation	92
	KLF5	Fbw7	BC	Cell proliferation	93
	ITCH	CFLARL	NSCLC	Apoptosis	82
	RNF4	PML-RARα	APL	Apoptosis	94
	GPX4	Fbw7	BC	Ferroptosis	11
	\	E2F-1	PANCAN	DNA repair	95
	UCHL3	TDP1	HCC	DNA repair	96
	VHL	KLF4	BC	Genomic stability	97
	β-Trcp	Mxi1	NSCLC	Drug resistance	98
	\	Rbfox2	GBM	Drug resistance	99
	TRIM28	ALKBH5	CRC	Immune escape	100
	ITCH	RORα	HCC	Tumor progression	101
	UBR5	ASCL4	RCC	Ferroptosis	102
PRMT6	TRAF6	EZH2	GBM	Invasion and migration	103
	\	c-MYC	CRC	Cell proliferation	104
	\	RBM39	NSCLC	Drug resistance	105
PRMT7	\	MRPS23	BC	Invasion and migration	106

This section systematically reviews the molecular mechanisms of protein methylation‒ubiquitination interactions in tumor biology, with particular emphasis on their regulatory networks and biological importance in critical pathological processes, including tumor invasion/metastasis, abnormal proliferation, ferroptosis regulation, programmed cell death, metabolic reprogramming, and immune microenvironment remodeling.

### Tumor invasion and metastasis

Recent studies have revealed the synergistic regulatory role of protein methylation and ubiquitination in tumor invasion and metastasis. The interaction mechanism between epigenetic modification enzymes and the ubiquitination system has attracted significant attention and remains the most extensively studied area. Various studies targeting key proteins involved in tumor metastasis have revealed multilevel mechanisms of coordinated action between methylation and ubiquitination, which can be categorized into the following types:

First, PRMT enzymes directly modify key effector proteins for metastasis by inhibiting their ubiquitination-mediated degradation. For example, the arginine methyltransferase PRMT1 mediates ADMA at the R342 site of EZH2 (enhancer of Zeste 2), suppressing CDK1 kinase-mediated phosphorylation at the T345/T487 sites of EZH2 through steric hindrance or conformational changes. This inhibits the binding of the E3 ubiquitin ligase TRAF6, enhances EZH2 stability, and promotes breast cancer (BC) metastasis.^79^ This finding suggests that methylations can achieve functional “reprogramming” by regulating protein modification states (phosphorylation) rather than mere stability. Arginine methyltransferase CARM1 (PRMT4) catalyzes dimethylation at the R210/R582 sites of TRIM47, antagonizing binding by the ubiquitin ligase CRL4 complex and inhibiting the ubiquitination-mediated degradation of TRIM47.^3^ Stabilized TRIM47 interacts with the transcription factor SNAI1 to block its proteasomal degradation, thereby driving HCC cell EMT and metastasis. This mechanism reveals how methylations “protect” ubiquitinated substrates, maintaining the functional activity of key metastasis proteins. In contrast, PRMT enzymes directly modify ubiquitin ligase binding sites to increase target protein stability. For example, CARM1 mediates dimethylation at the R838 site of the histone demethylase LSD1, promoting binding between the ubiquitin ligases USP7 and LSD1, thereby inducing LSD1 ubiquitination and stabilizing it. Stabilized LSD1, which functions as a histone demethylase, drives breast cancer (BC) invasion and metastasis by regulating the expression of metastasis-associated genes (e.g., EMT transcription factors).^89^ This mechanism establishes methylation as a molecular bridge connecting ubiquitin ligases with target proteins, forming a positive regulatory circuit of “methylation-ubiquitination-stabilization”.

Additionally, PRMT enzymes indirectly regulate the ubiquitination of target proteins by suppressing ubiquitin ligase transcription through histone methylation. For example, the arginine methyltransferase PRMT6 induces ADMA at the H3R2 site (H3R2me2a), inhibiting TRAF6 gene transcription. Reduced TRAF6 expression weakens its protein interaction with EZH2, decreasing the degradation of ubiquitinated EZH2 and increasing its stability. Stable EZH2 promotes glioblastoma (GBM) invasion and metastasis by regulating downstream metastasis-related gene expression.^103^ Furthermore, coordinated methylation–ubiquitination regulation modulates mitochondrial function and tumor metastasis through metabolic reprogramming. The arginine methyltransferase PRMT7 and the lysine methyltransferase SETD6 modify the R21 and K108 sites, respectively, of the mitochondrial ribosomal protein MRPS23. R21 methylation accelerates the degradation of MRPS23 via the polyubiquitination pathway, maintaining its low expression level. The degradation of MRPS23 inhibits oxidative phosphorylation (OXPHOS), thereby reducing the production of mitochondrial reactive oxygen species (mtROS).^106^ As mtROS serve as critical stress signals that drive tumor metastasis—for example, by inducing EMT and promoting angiogenesis—the PRMT7-SETD6-MRPS23 axis regulates metabolic reprogramming (by suppressing OXPHOS) to diminish the metastatic potential of tumor cells.

### Tumor proliferation

The infinite proliferation of cells is a core characteristic of malignant cancers, and significant progress has been made in the study of its molecular mechanisms. The role of the coordinated regulatory network of epigenetic modifications and posttranslational modifications in protein translation in cancer proliferation has gradually been elucidated. PRMT enzymes mediate arginine methylation to regulate substrate stability through two primary mechanisms that promote tumor growth. The first mechanism involves PRMT enzymes that methylate substrates to recruit deubiquitinases, thereby removing ubiquitination marks and reducing their degradation via the proteasome. For example, the methyltransferase CARM1 methylates the R348 site of O-linked N-acetylglucosamine transferase (OGT), enhancing OGT binding with USP9X and stabilizing OGT. This increases O-GlcNAcylation levels, which enhances the glycolytic capacity of non-small cell lung cancer (NSCLC) cells.^90^ PRMT1 methylates the R127 site of cGAS, recruiting USP7 to deubiquitinate cGAS and maintain its stability. cGAS then activates the AKT signaling pathway, accelerating NSCLC cell proliferation.^80^

The second mechanism involves PRMT enzymes methylating substrates to reduce their affinity for ubiquitin ligases, blocking the ubiquitination process and thereby decreasing substrate degradation. For example, the arginine methyltransferase PRMT5 methylates the GLI family zinc finger 1 (GLI1) transcription factor, reducing its binding to the ubiquitin ligase ITCH/NUMB. This leads to abnormal accumulation of GLI1 within the nucleus and sustained activation of the Hedgehog (HH) signaling pathway, thereby increasing tumor proliferation.^92^ Additionally, PRMT5 methylates the R57 site (R57me2) of the transcription factor KLF5, antagonizing phosphorylation mediated by the serine/threonine protein kinase GSK3β and blocking ubiquitination degradation by the E3 ubiquitin ligase Fbw7.^93^ This mechanism is crucial for maintaining the self-renewal capacity of BC stem cells. Similarly, in CRC, PRMT6 methylates the R371 site of the oncogene c-MYC, inhibiting its polyubiquitinated degradation and promoting tumor cell proliferation.^104^ In HCC models, PRMT1 catalyzes NADP-dependent malonate enzyme 2 (ME2) methylation. By suppressing the interaction of ME2 with Fbw7 and reducing its ubiquitination-mediated degradation, this process enhances the malignant proliferation of liver cancer cells.^81^

## Cell death

### Apoptosis

Apoptosis, a fundamental form of programmed cell death, is of considerable biological importance in cancer treatment. The interplay between methylation and ubiquitination in epigenetic modifications has been shown to influence the regulation of apoptosis in cancer cells. Researchers led by Li et al revealed that PRMT1 and PRMT5 regulate the degradation of the antiapoptotic protein CFLARL through antagonistic effects between ADMA and SDMA, thereby activating caspase-8-dependent apoptosis pathways.^82^ Another regulatory mechanism involves direct modification of key components in the ubiquitination system by PRMT family enzymes. A typical example is that arginine methylation at USP9X significantly enhances its protein interaction with TDRD3, thereby strengthening the antiapoptotic activity of TDRD3 in BC cells.^107^ In acute promyelocytic leukemia (APL) models, PRMT5-mediated methylation at the Arg164 site of the PML-RARα protein significantly inhibits the binding stability between the oncogene protein and its ubiquitin E3 ligase RNF4 through steric hindrance effects, ultimately affecting APL cell differentiation and apoptosis processes.^94^

### Ferroptosis

Ferroptosis, a prominent area of research in cancer biology in recent years, has attracted increasing attention for its regulatory mechanisms and interactions with epigenetic modifications.^108^, ^109^, ^110^ Numerous studies have demonstrated that the molecular crosstalk between methylation and ubiquitination plays a crucial role in the regulation of ferroptosis. The PRMT enzyme family mediates substrate protein methylation to positively or negatively regulate downstream ubiquitination processes through two primary mechanisms. The first involves methylation, which inhibits substrate ubiquitination to increase stability. For example, PRMT5 methylates GPX4 (a key inhibitor of ferroptosis), preventing its binding to the Cullin1-FBW7 E3 ligase complex. This reduces ubiquitination degradation rates and strengthens GPX4 stability, thereby weakening tumor cell resistance to ferroptosis.^11^ The second mechanism involves methylation, which promotes substrate ubiquitination to accelerate degradation. A representative example is PRMT5-mediated symmetrical dimethylation of the arginine residues in ACSL4, which facilitates its binding to the ubiquitin ligase UBR5.^102^ This interaction decreases ACSL4 stability and suppresses ferroptosis in renal cell carcinoma (RCC) cells. Similarly, CARM1 methylates the R339 site of ACSL4 (a key promoter of ferroptosis), enhancing its binding to the E3 ligase RNF25. This facilitates ACSL4 ubiquitination and degradation, ultimately inhibiting ferroptosis.^91^

### DNA repair and genome stability

Abnormalities in DNA repair and genomic instability are hallmark features of malignant tumor cells.^111^ The interplay between methylation and ubiquitination plays a crucial role in maintaining tumor DNA repair and genomic stability, with methylation primarily influencing this process by regulating the homeostasis of repair proteins.

For example, PRMT5 disrupts the proteasomal degradation pathway of E2F-1 by modifying its biochemical properties or competitively displacing its binding partners. Notably, under DNA damage conditions, the arginine methylation level of E2F-1 decreases significantly, increasing its structural stability and promoting DNA repair.^95^ In liver cancer models, PRMT5 catalyzes R361/R586 methylation of tyrosyl DNA phosphatase 1 (TDP1). R586 methylation blocks the binding of the ubiquitination ligase UCHL3, facilitating the degradation of TDP1 via ubiquitination and regulating Top1 cc-mediated repair to maintain genomic stability.^96^ Additionally, in BC models, PRMT5 mediates KLF4 arginine methylation by inhibiting VHL (E3 ubiquitin ligase)-mediated ubiquitination. This reduction in the KLF4 metabolic rate enhances the transcription of KLF4-dependent cyclin p21 (to suppress proliferation) and inhibits the Bax protein (to promote apoptosis), thereby maintaining genomic stability.^97^

### Cancer metabolic reprogramming

Cancer metabolic reprogramming, a critical pathological characteristic of malignant tumors, involves the precise coordination of methylation and ubiquitination.^112^ Methylation regulates the stability of metabolism-related proteins, thereby promoting tumor metabolic reprogramming. This epigenetic regulatory axis exerts decisive control over multidimensional metabolic biological processes during tumorigenesis.

In non-alcoholic steatohepatitis (NASH) models, PRMT1 catalyzes arginine methylation on the apoptosis regulator CFLAR (CASP8 and FADD-like apoptosis regulator), triggering its ubiquitinated degradation. This inhibition of downstream activated protein kinase JNK signaling pathways leads to hepatic lipid metabolism imbalance, facilitating NASH progression to HCC.^84^ Additionally, PRMT1 induces R578 methylation of the RNA-binding protein RBM15, promoting its degradation via the E3 ligase CNOT4.^85^ The subsequent reduction in RBM15 stability enables splicing regulation through its interaction with introns of target genes and recruitment of the SF3B1 splice factor complex. Abnormal splicing of myeloid leukemia-related genes results from this degradation process.

### Drug resistance

Drug resistance in cancer treatment remains a major challenge in clinical oncology, as its mechanisms are closely linked to epigenetic modifications and ubiquitination regulation. The interaction between arginine methylation and ubiquitination forms a critical molecular axis of tumor resistance. This methylation‒ubiquitination interaction mediates downstream resistance-related events by regulating the stability of key proteins. Specifically, PRMT1 catalyzes arginine methylation on nucleolar and spindle-associated protein 1 (NUSAP1). The modified NUSAP1 specifically binds to the PEST domain of Notch receptor 2 (Notch2) through its methylation site, blocking the interaction of Notch2 with ubiquitin ligases and inhibiting its ubiquitination-mediated degradation, thereby maintaining protein stability. As Notch2 is a classic oncogene, its stabilized state perpetuates Notch pathway activation, promoting gastric cancer (GC) cell proliferation and antiapoptotic capacity, ultimately leading to 5-FU chemotherapy resistance.^86^ Moreover, PRMT6 catalyzes R92-arginine methylation on RNA-binding motif protein 39 (RBM39). Methylated RBM39 cannot be induced by the targeted splicing factor drug indisulam to bind with ubiquitin ligases, thus suppressing its ubiquitination and proteasomal degradation, resulting in abnormal accumulation of RBM39. This abnormal phenomenon drives the selective splicing of proto-oncogenes (e.g., EGFR and ALK), generating oncogenic isoforms that contribute to NSCLC resistance to targeted therapies.^105^

The aforementioned process demonstrates that arginine methylation inhibits ubiquitination-mediated degradation, thereby leading to abnormal activation of downstream drug resistance-related signaling pathways or disordered alternative gene splicing. Additionally, arginine methylation promotes ubiquitination-mediated degradation, accelerating substrate clearance and causing an imbalance in protein homeostasis, which affects cellular drug resistance. PRMT5 catalyzes arginine methylation on MAX Interactor 1 (Mxi1), which increases its binding affinity with the ubiquitin ligase β-tricoxonate (Tricor) and accelerates its ubiquitination-mediated degradation, resulting in reduced protein levels. As a negative regulatory factor for the proto-oncogene c-Myc, ubiquitinated Mxi1 weakens its inhibitory effect on c-Myc, activating the c-Myc pathway and enhancing radioresistance in NSCLC cells.^98^

Furthermore, specific types of ubiquitination regulate substrate functions and influence the drug resistance process. For example, PRMT5 catalyzes arginine dimethylation on RNA-binding Fox-1 homology 2 (Rbfox2). The modified Rbfox2 is recognized by the ubiquitin protein FBXO7 and undergoes K63-linked ubiquitination. This ubiquitination does not mediate degradation but instead enhances Rbfox2 protein stability. Stabilized Rbfox2 regulates the splicing of mesenchymal genes (such as vimentin and Snail), promoting EMT in GBM.^99^

### Immune escape mechanisms

The immune escape mechanism, a critical area of cancer research, has garnered significant attention because of its regulatory relationship with the crosstalk between methylation and ubiquitination.^113^^,^^114^ Methylation regulates immune checkpoint molecule expression through core processes mediated by the PRMT family, specifically protein methylation and crosstalk with ubiquitination. By modulating the stability of key intermediate molecules, this mechanism ultimately drives the abnormal expression of immune checkpoint molecules, thereby promoting tumor immune evasion.

For example, in CRC models, PRMT5 directly catalyzes SDMA (meR316-ALKBH5) at the R316 site of the RNA demethylase ALKBH5. This modification significantly reduces ALKBH5 protein stability by enhancing the ubiquitination degradation pathway mediated by the E3 ubiquitin ligase TRIM28. Mechanistic studies demonstrated that decreased ALKBH5 levels weaken 3′ nontranslational region m6A demethylation of CD276 mRNA, a T-cell activation-associated immunoglobulin, leading to increased mRNA stability and abnormally high expression in CRC cells. *In vitro* and *in vivo* experiments confirmed that CD276 up-regulation significantly accelerates CRC immune evasion by impairing cytotoxic T-cell function. Further research revealed that PRMT5-mediated meR316-ALKBH5 modification drives CRC immune evasion at the molecular level by activating CD276 transcript m6A modification mechanisms.^100^

In nasopharyngeal carcinoma (NPC) models, Epstein–Barr virus (EBV)-encoded latent membrane protein 1 (LMP1) induces peroxisome proliferator-activated receptor-γ coactivator-1α (PGC-1α) to undergo methylation by enhancing its interaction with PRMT1 and PGC-1α. This inhibition of proteasome-mediated degradation significantly improved protein stability. Furthermore, PGC-1α forms a coactivation complex with STAT3, up-regulating PD-L1 expression to induce T-cell depletion, thereby mediating LMP1-induced immune evasion. Additionally, PGC-1α enhances the antiapoptotic capacity of NPC cells through the regulation of mitochondrial function, further supporting this immune evasion process.^87^

### Tumor development

Understanding the molecular mechanisms underlying cancer development is essential for cancer research, particularly regarding the interplay between methylation and ubiquitination. The progression of tumors involves a multistep, multifactorial process in which coordinated actions between epigenetic modifications (e.g., methylation) and protein function regulation (e.g., ubiquitination) are pivotal. Arginine methyltransferases (PRMTs) modify target proteins through arginine methylation, altering their conformation or interacting with E3 ubiquitin ligases to regulate their ubiquitination status (type or level), ultimately influencing downstream tumor-related signaling pathways.

For example, PRMT1 can methylate two arginine residues on the E3 ubiquitin ligase RACO-1. This modification induces conformational changes in RACO-1, significantly promoting the formation of K63-type ubiquitin chains and enhancing its protein stability. Subsequent studies revealed that conformational changes in RACO-1 constitute the structural basis for its regulation of ubiquitination.^88^ When gene editing eliminates PRMT1 function, the activity of the transcription factor AP-1 is markedly impaired, leading to approximately 78% down-regulation of c-Jun-dependent mitogen-activated protein kinase (MAPK) target genes. This study demonstrated that arginine methylation of RACO-1 is essential for the transcriptional activation function of the classic oncogenic c-Jun/AP-1 pathway. PRMT1 stabilizes RACO-1 to activate this pathway, thereby exerting its oncogenic effects.

Furthermore, PRMT5 dynamically regulates the interaction between E3 ubiquitin ligases (ITCHs) and RORα by modifying their arginine methylation status. This process catalyzes the formation of K48-type ubiquitin chains (a classic degradation signal), leading to RORα degradation via the proteasome pathway and a significant reduction in protein levels. The degradation of RORα mediated by PRMT5 blocks downstream target gene activation, ultimately exerting tumor suppressive effects on HCC cells and thereby regulating cancer cell fate.^101^

### Regulation of methyltransferases by the USP system

There are bidirectional interactions between methylation and ubiquitination regulatory networks. The ubiquitination–deubiquitination system achieves multidimensional regulation of epigenetic modifications by modulating methyltransferase stability, enzyme activity, and complex assembly. An imbalance in this system serves as a key driver of tumor development, metabolic reprogramming, and drug resistance.

Most ubiquitination systems (particularly E3 ubiquitin ligases) directly regulate protein stability by mediating the ubiquitination of methyltransferases. For example, the E3 ubiquitin ligase TRIM21 inhibits CRC cell proliferation, tumor formation, migration, and metastasis by promoting the degradation of ubiquitin-dependent PRMT1.^6^ The E3 ubiquitin ligase FBXO7 significantly enhances proteasome-dependent degradation efficiency by inducing ubiquitination at lysine 37 (Lys37) in PRMT1, leading to metabolic homeostasis disruption and proliferation inhibition in HCC.^78^ Additionally, some E3 ligases stabilize complexes with cofactors by catalyzing the ubiquitination of methyltransferases, thereby increasing their activity. For example, the E3 ubiquitin ligase TRAF6 promotes the ubiquitination of PRMT5, increasing H4R3me2s levels, which facilitates malignant proliferation in BC.^119^

Conversely, deubiquitinases primarily maintain protein stability by removing ubiquitination modifications from methyltransferases. For example, the ubiquitin ligase USP7 binds to PRMT1 under glucose-rich conditions, preventing its ubiquitination-mediated degradation and thereby promoting aerobic glycolysis in NSCLC cells (through the PRMT1-PTBP1-PKM2 pathway).^117^ The ubiquitin ligase PSMD14 maintains PRMT4 stability by removing its ubiquitination, driving HCC proliferation via the PRMT4-H3R17me2a-FERMT1 pathway.^118^ Additionally, ubiquitin ligases regulate biological functions by modulating the assembly of methyltransferases and downstream signaling complexes. For example, USP11 forms an interacting complex with PRMT1 to regulate the activity of the PRMT1-MRE11 signaling pathway, participating in early-stage methylations during DNA damage repair.^5^

The functional status (activity/degradation) of methyltransferases is determined by the balance between ubiquitination (by E3 ligases) and deubiquitination (by DUBs). For example, PRMT1 stability is jointly regulated by CHIP (degrading) and USP7 (stabilizing): under glucose deficiency, p53 inhibits USP7 expression, leading to CHIP-mediated PRMT1 degradation, which suppresses glycolysis^115^; under high glucose conditions, USP7 binds to PRMT1, stabilizing its expression and promoting glycolysis.^117^ This equilibrium mechanism ensures cellular adaptability across different microenvironments, but imbalances (such as overexpression of USP7 or downregulation of CHIP) can lead to excessive activation of methyltransferases, driving tumor development. Furthermore, the ubiquitination–deubiquitination system regulates methyltransferases with spatiotemporal specificity. For example, FBXO7 expression is significantly down-regulated in liver cancer tissues, leading to reduced degradation and increased stability of PRMT1, which drives metabolic imbalance in HCC.^78^ Conversely, TRIM21 is highly expressed in CRC, where it promotes PRMT1 degradation and inhibits CRC proliferation.^6^ This tissue specificity may be related to the tumor microenvironment (TME), such as metabolic status and immune cell infiltration. HCC cells rely on glycolysis, where stable PRMT1 facilitates metabolic reprogramming, whereas CRC cell proliferation depends more strongly on PRMT1 inhibition, thus activating TRIM21.^6^ Additionally, tissue-specific expression of E3 ligases and DUBs (such as FBXO7, which is down-regulated in liver cancer) is another key factor contributing to regulatory differences.

Notably, posttranslational modifications of methyltransferases can dynamically regulate their ubiquitination processes. Yuan et al revealed that PRMT5 K387 deacetylation mediated by the deacetylase SIRT7 effectively reduces ubiquitination levels mediated by the E3 ligase STUB1 and enhances methyltransferase activity.^121^ This subsequently drives lipid metabolism reprogramming in HCC through stabilizing the expression of SREBP1a, a protein associated with lipid synthesis. This “modification crossover” means that methyltransferase functions are not only regulated by the ubiquitination system but also influenced by metabolic states (e.g., succinyl-CoA levels). For example, HCC cells exhibit active lipid metabolism with elevated succinyl-CoA levels, which may promote the succinylation of PRMT5 and thereby increase its ubiquitination-mediated degradation. Conversely, the overexpression of SIRT7 removes this succinylation, stabilizing PRMT5 and driving lipid metabolism reprogramming. This “cross-modification” mechanism further highlights the complexity of methyltransferase regulation.

However, given the limited data available, only examples of deubiquitinases regulating PRMT1, PRMT4 and PRMT5 have been identified (Table 2), and there is still much room for research in this field.

**Table 2:** Ubiquitinase regulates methyltransferase stability.

(De)ubiquitinating enzymes	PRMT enzyme	Effect	cancer	Functions	Ref
TRIM21	PRMT1	Degradation	CRC	Tumor progression	6
CHIP	PRMT1	Degradation	Osteosarcoma	Apoptosis	115
TRIM48	PRMT1	Degradation	HCC	Oxidative stress	116
USP7	PRMT1	Stabilization	NSCLC	Cell proliferation	117
USP11	PRMT1	Stabilization	BC	DNA repair	5
FBXO7	PRMT1	Degradation	HCC	Cell proliferation	78
PSMD14	PRMT4	Stabilization	HCC	Invasion and migration	118
TRAF6	PRMT5	Activation	BC	Cell proliferation	119
UBR7	PRMT5	Degradation	PDAC	Drug resistance	120
STUB1	PRMT5	Degradation	HCC	Metabolism reprogramming	121

## Conclusions, challenges, and prospects

This study systematically elucidates the interactive regulatory network of arginine methylation and ubiquitination in cancer development, revealing the synergistic mechanisms of DNA methyltransferases and E3 ubiquitin ligases at the epigenetic level. The crosstalk between arginine methylation and ubiquitination forms dynamic molecular switches that mediate epigenetic reprogramming and disrupt protein homeostasis, constituting a core regulatory hub in cancer development. This bidirectional regulatory network influences the cancer cell cycle and genomic stability by modulating key signaling pathways at the molecular level.

Current research on the crosstalk network between arginine methylation and ubiquitination faces three significant challenges. First, the bidirectional regulatory network involving PRMT enzymes and ubiquitin ligases/deubiquitinases demonstrates spatiotemporal specificity.^5^ This complexity complicates the ability of existing small-molecule inhibitors to accurately target the conformational states of enzyme activity under specific pathological conditions.^122^ This process necessitates the application of structural analysis and computational simulation methods to screen conformation-dependent compounds. Additionally, the combination of epigenetic drugs and proteasome inhibitors may result in a toxic superposition effect characterized by cross-interference between epigenetic reprogramming and an imbalance in protein homeostasis.^123^ Therefore, a selective regulatory strategy is essential. Third, the technology for monitoring dynamic modifications is currently inadequate, and there is a deficiency of multiomics tracking methods at the single-cell level for the protein degradation process mediated by the interplay of PRMTs and E3 ligases.^124^ This situation necessitates the development of a new monitoring system that integrates dynamic modification detection with real-time metabolic analysis.

Future research should concentrate on the intersection of emerging cellular processes and the methylation‒ubiquitination network. Specifically, arginine methylation of copper death-related proteins may affect their ubiquitin-dependent mitochondrial localization by modulating the activity of E3 ubiquitin ligases.^125^^,^^126^ This dynamic modification balance could be mapped via CRISPR screening technology to create a regulatory map of methyltransferases and deubiquitinases.^127^^,^^128^ In addition, the roles of methyltransferases and ubiquitin enzymes in phase separation droplets remain unclear, and the microenvironment of phase transitions may introduce a new dimension for regulating interactions.^129^^,^^130^ It is essential to employ cryo-electron microscopy and fluorescence resonance energy transfer (FRET) techniques to analyze the characteristics of enzymatic reactions at the phase interface.^131^

Additionally, the dynamic interplay between PRMT enzyme-mediated methylation and the ubiquitin system may collaboratively regulate the stability and function of immune checkpoint proteins. This interaction provides multidimensional intervention targets, including small-molecule degraders and engineered nanobodies, to overcome resistance to immunotherapy.^132^, ^133^, ^134^, ^135^, ^136^ Future research should concentrate on the spatiotemporal specificity of the PRMT-ubiquitin cross-regulation network in immune evasion. Additionally, dual-function inhibitors that target both epigenetic modifiers and ubiquitin proteases should be developed, and their synergistic enhancement mechanisms in conjunction with existing immune checkpoint blockade therapies should be investigated. Importantly, the spatial coupling mechanism of the methylation gradient within the mitochondrial intermembrane space, in relation to cytoplasmic ubiquitination signals, may emerge as a pivotal research paradigm that connects cell fate determination with metabolic homeostasis.^137^^,^^138^

This article presents numerous examples of crosstalk between arginine methylation and ubiquitination. However, the interactions between specific types of arginine methyltransferases and ubiquitinases remain underexplored, necessitating further research to enrich this field. In summary, the interplay between arginine methylation and ubiquitination plays a pivotal role in cancer treatment. Arginine methylation regulates protein stability, activity, and localization during critical processes, such as cell cycle control, DNA damage repair, and apoptosis, which are all essential for cancer progression and therapeutic responses. Conversely, ubiquitination mediates protein degradation, affecting arginine-methylated proteins and other core signaling proteins that regulate cancer cell survival and death. The delicate balance between these two mechanisms is often disrupted in cancers, exacerbating tumor progression. Current studies have investigated how arginine methylation influences ubiquitination primarily to explore its role in protein stability and function, while ubiquitination itself can exert feedback regulation by altering arginine methylation levels. Targeting enzymes involved in both arginine methylation and ubiquitination may become novel strategies to overcome cancer treatment bottlenecks. Additionally, a deeper understanding of the interaction network between these two modification pathways could pave the way for the development of novel therapies that target cancer cells.

## CRediT authorship contribution statement

**Jiawen Zhou:** Writing – original draft, Resources, Conceptualization. **Ao Zhang:** Validation, Conceptualization. **Jiuling Zhu:** Methodology, Conceptualization. **Fei Tang:** Resources. **Ziyang Yuan:** Resources. **Wenlong Ma:** Resources. **Qi Wang:** Investigation. **Jun Lu:** Writing – review & editing, Supervision. **Shu Li:** Writing – review & editing, Supervision. **Zhongwei Li:** Writing – review & editing, Writing – original draft, Validation, Supervision, Project administration, Investigation.

## Funding

This work was supported by grants from the Nature Science Foundation of Jiangsu Province, China (No. 82173060, 82573790); the Outstanding Youth Project of the University Natural Science Research in Anhui Province, China (No. 2024AH020013); the Key Project of the University Natural Science Research in Anhui Province, China (No. 2023AH051774); the Launch Foundation for High Level Talent Research of Wannan Medical University (China) (No. WYRCQD2024011); the Wannan Medical University Doctoral Research Startup Fund (China) (No. WYRCQD2022005); the Wannan Medical University Student Research Grant (China) (No. WK2024XS58).

## Conflict of interests

No potential conflict of interests was disclosed by the authors.
